# The future of transcranial ultrasound as a precision brain interface

**DOI:** 10.1371/journal.pbio.3002884

**Published:** 2024-10-29

**Authors:** Keith Murphy, Elsa Fouragnan

**Affiliations:** 1 Department of Radiology, Stanford University, Stanford, California, United States of America; 2 Attune Neurosciences, San Francisco, California, United States of America; 3 Brain Research and Imaging Centre, University of Plymouth, Plymouth, United Kingdom; 4 School of psychology, Faculty of Health, University of Plymouth, Plymouth, United Kingdom

## Abstract

Our understanding of brain circuit operations and disorders has rapidly outpaced our ability to intervene and restore them. Developing technologies that can precisely interface with any brain region and circuit may combine diagnostics with therapeutic intervention, expediting personalised brain medicine. Transcranial ultrasound stimulation (TUS) is a promising noninvasive solution to this challenge, offering focal precision and scalability. By exploiting the biomechanics of pressure waves on brain tissue, TUS enables multi-site targeted neuromodulation across distributed circuits in the cortex and deeper areas alike. In this Essay, we explore the emergent evidence that TUS can functionally test and modify dysfunctional regions, effectively serving as a search and rescue tool for the brain. We define the challenges and opportunities faced by TUS as it moves towards greater target precision and integration with advanced brain monitoring and interventional technology. Finally, we propose a roadmap for the evolution of TUS as it progresses from a research tool to a clinically validated therapeutic for brain disorders.

## Abstract 2

Transcranial ultrasound stimulation (TUS) is a promising non-invasive solution to interface with specific brain regions with high precision to combine diagnostics with therapeutic interventions. This Essay explores the challenges and proposes a roadmap for the evolution of TUS from a research tool to a clinically validated therapeutic.

## Introduction

Psychiatric, neurological, and developmental brain disorders are mediated by changes in the structure and function of brain circuits and have an overwhelming societal and economic burden affecting one in 4 individuals worldwide [1]. While methods to identify and map structural abnormalities in the brain circuits exist, mapping of functional abnormalities is far more complex. Furthermore, the mapping of functionally aberrant circuits is only the first hurdle since current technologies for precise brain intervention involve highly invasive implants or focal lesions which lack potential to impact the masses [2]. We believe that a noninvasive and reversible neuromodulation technology with high spatial resolution can address these shortcomings: to perturb-and-measure for assessing brain circuit function, and to perform spatially informed interventions. In principle, such a technology could be applied to all brain disorders, unifying and significantly advancing neuromodulation solutions at scale.

Over the last several decades, major advances have been achieved in identifying and mapping both micro- and macro-structural abnormalities in the brain, especially in the context of neurological disorders. Magnetic resonance imaging (MRI), for example, allows for the clear visualisation of neural anatomy at a resolution of approximately one cubic millimetre [3]. Leveraging machine learning and large scale, high-resolution quantitative measures of the brain allow early prediction and treatment planning for conditions like Parkinson’s disease or essential tremor [4]. These advances include MR fingerprinting techniques, which generate unique tissue identifiers for improved characterisation of brain matter [5] that are beginning to be adopted by clinical practitioners [6]. In addition to changes in structure, brain disorders also arise from changes in functional connectivity of neural circuits, loosely described by changes in information flow between discrete regions of the brain [7]. These functional changes lead to maladaptive cognitive states and behaviours but may be corrected by modifying input–output relationships across specific regions. While this concept holds true in theory, our understanding of the exact link between brain regions and symptoms is still insufficient to establish a panacea. In fact, individuals can display similar symptoms despite major differences in underlying network disorders [8], undermining the utility of classic symptomatology. Accordingly, treatment of any given network node will not necessarily benefit each patient of a symptomatic class, evidenced by the variability in effective sites of deep brain stimulation (DBS) symptom relief in major depressive disorder [9]. Although these network differences have been challenging to pinpoint, advanced neuroimaging methods have improved our ability to identify them. For instance, functional MRI (fMRI) is used to track blood oxygenation levels across the brain as a proxy for neural activity and can be used to monitor the amount of activity in a given area, or its correlation with other areas. Differences in these absolute levels or cross-brain correlations from healthy controls can suggest sites in need of treatment. Additionally, computational models of cognitive processes and psychiatric traits mapped onto brain circuits at a system level may inform therapeutic interventions [10–12]. However, to refine these models and create predictive models of disease, cognitive neuroscience needs causal investigations of the circuits at play through precise neuromodulation.

Neuromodulation can be achieved using several techniques, including DBS—involving implanting electrodes to stimulate specific brain regions—transcranial magnetic stimulation (TMS), which uses magnetic fields to noninvasively stimulate largely superficially located cortical areas, and transcranial direct current stimulation (tDCS), which applies weak electrical currents through scalp electrodes to modulate neuronal excitability. However, these techniques have inherent limitations. DBS, while effective for certain conditions, requires invasive surgery and carries risks such as infection and hardware complications. Conversely, although TMS and tDCS are noninvasive, they lack the same level of precision, may cause discomfort, and are limited to lateral surfaces. When combined with neuroimaging methods such as MRI and fMRI, these techniques help map brain activity and monitor changes; however, they introduce additional challenges. For instance, MRI can be unsafe for DBS patients due to metal implants, and combining MRI with TMS or tDCS is limited by interference and compatibility issues, which can distort imaging data and pose safety risks, complicating the precise mapping of brain activity [13,14].

A promising alternative noninvasive treatment that aims to overcome these limitations is transcranial focused ultrasound (TUS), which creates highly focused ultrasound fields through the skull within the brain, altering neural regions that are responsive to acoustic energy [15,16]. By showing that TUS can safely induce neural activity changes in the brain with high spatial resolution and with the potential to bias responses for specific cell types and brain areas [17–20], TUS has become one of the most promising and potentially revolutionary precision neuromodulatory technologies of the last decade.

## Mechanisms, effects, and resolution of TUS

With the growing enthusiasm for TUS applications, mechanisms of low intensity focused ultrasound neuromodulation are being actively investigated at the molecular, cellular, and system levels [21,22]. Although there is still active debate as to which TUS biophysical effect drives neuromodulation, a fundamental link between physical displacement, temperature, and neuronal activity is well understood. TUS induces mechanical displacement of cell membranes through either the cyclical compression with each passing wave or cumulative net distortion. The cyclical compression adds kinetic energy to the membrane as it oscillates around its starting position. The cumulative distortion, also known as radiation force displacement, occurs as a result of absorption and would appear as a stable distortion of the membrane for as long as a pulse is active, observed as energy absorbed by the brain tissue [15]. Additionally, mechanical strain is known to change conductance across a myriad of ion channels classes [23,24]. Although the exact mechanism by which mechanical force opens ion channels is still under investigation [25,26], one line of evidence points to increased biochemical reactions through release of organised lipid rafts containing enzymes. These “release enzymes” interact with nearby substrates and the enzymatic product of these reactions rapidly opens ion channels [27,28]. Beyond compressional force, shear force is also induced as brain fluids, such as cerebrospinal fluid and blood, flow upon ultrasound application, a phenomena known as acoustic streaming [29]. The resulting shear forces of this flow likely engage mechanosensors and modulate tissue function. For instance, blood vessel endothelial cells can sense this flow and will dilate or contract in response, which may further impact neural activity through changed availability of nutrients and blood-borne messengers, as well as changes in temperature and oxygen availability [30–32].

In addition to mechanical effects, the influence of temperature on neuronal activity is well evidenced and incorporated into models of resting membrane potential and membrane conductance [33,34]. Since focused ultrasound waves deposit heat through friction, it logically follows that TUS can alter neuronal activity through temperature-induced changes in membrane properties. While the magnitude and directionality of this relationship can vary across cell types and brain areas, many studies have shown that even minor temperature changes can have effects on neuronal activity, heavily evidenced by heating confounds in mouse optogenetic experiments [35–38]. In support of this notion, peripheral nerve excitability was found to change with temperature increases as small as 1°C [39]. Importantly, temperature rise will depend heavily on TUS protocol intensity, duration, and tissue type. Together, these mechanisms provide a first principle understanding of how ultrasound mechanisms can collectively modulate neural activity.

**Fig 1:**
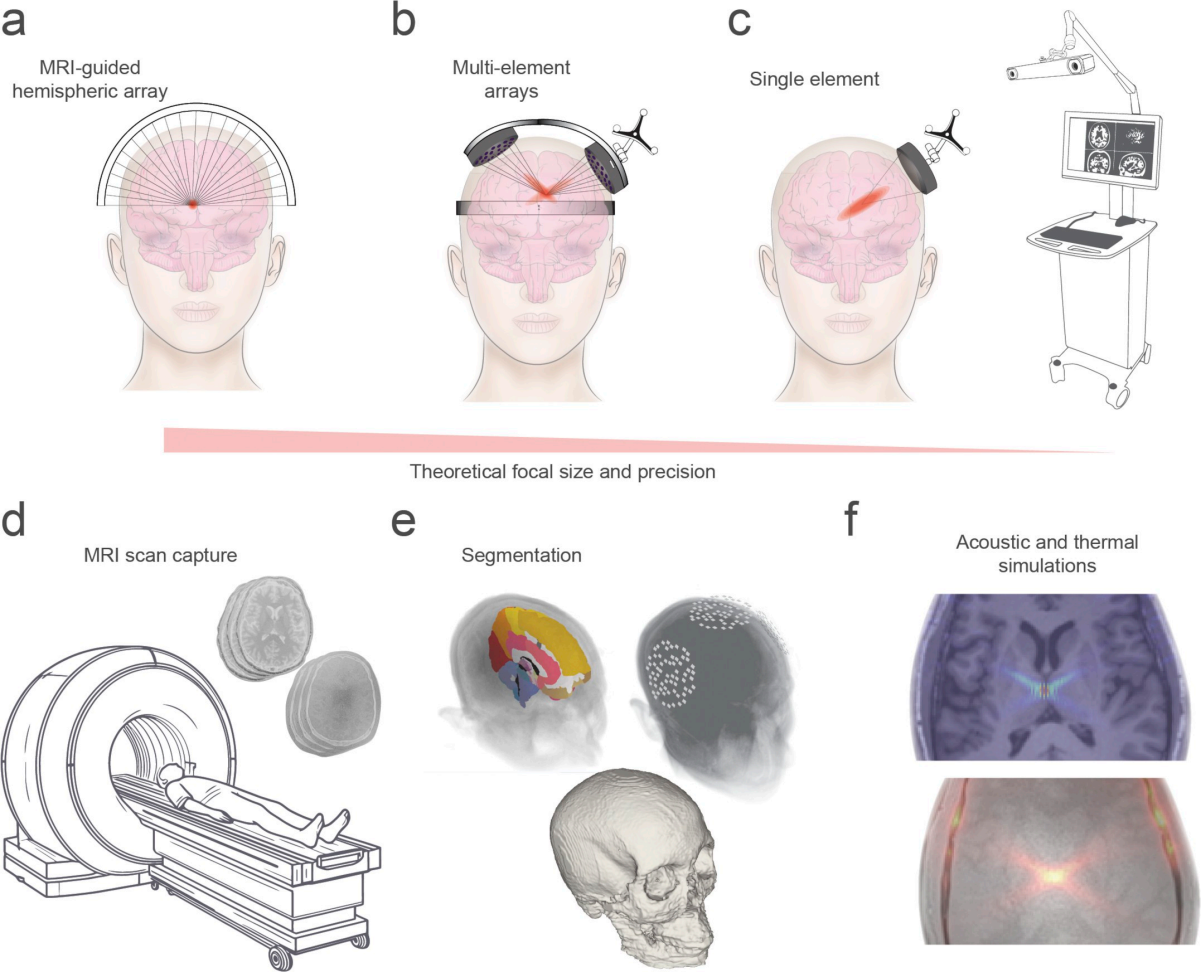
Focused ultrasound device selection and stimulation planning. Graphic illustrations of (**a**) an ultrasound array hemisphere, (**b**) 2 multielement transducers, and (**c**) a single-element transducer. (**d**) MRI capture of a brain anatomy through T1 imaging and skull density approximation through ultrashort echo time imaging. (**e**) Various stages of image segmentation prior to (**f**) acoustic simulation of waveform focusing in cranial space. MRI, magnetic resonance imaging.

TUS fields are often described by the volume of tissue that is exposed to a minimum pressure or intensity, although the relationship between biological effects and pressure or intensity is not known [18]. Larger TUS systems can achieve spatial scale and resolution as fine as a 1 mm sphere within the brain [40,41], far exceeding those of other noninvasive brain stimulation techniques. This configuration uses a large hemisphere containing multiple elements. With MRI guidance, this method allows for intricate control over the direction, timing, and consequently the focusing of ultrasound waves (Fig 1A). In addition to precision, these arrays provide the flexibility to steer a focal point throughout the brain, simply by adjusting the timing of each element’s activation. However, MRI-guided solutions usually necessitate that participants or patients shave their heads, and the procedure must be conducted within an MRI scanner. More versatile equipment, like independent multielement arrays can be used outside of the MRI scanner through optical device tracking and can implement a steerable focus to create more intricate patterns such as crossbeams (Fig 1B). While these devises are small enough to be manually placed anywhere on the head and offer large brain coverage, skull heating may be higher than other devices as it will depend on the distribution of source pressure on the radiating surface. For unique and more complex structural shapes or multiple targets, techniques like holographic rendering (precise 3D patterns of ultrasound waves) and raster scanning, which involves moving a focus beam in a grid-like pattern across targeted regions for neuromodulation, can effectively “paint” the brain with ultrasound energy, ensuring thorough coverage and precise modulation of neural activity. Importantly, while larger devices with multiple elements can provide superior spatial resolution, even transducers with a single or few elements may be capable of considerable precision (Fig 1C) [42,43]. Furthermore, planning stimulations with these transducers can be more accessible to researchers as the acoustic field will be easier to characterise [44–46].

For both the safety and accuracy of TUS treatment, acoustic wave propagation is simulated to estimate pressure and temperature evolution across the exposed cranial tissue ultrasound [42,44]. A crucial initial step is the acquisition of brain and skull images (Fig 1D), which enables the labelling of skull bone and acoustic property assignment of density, speed of sound, and attenuation. Another set of images allows anatomical mapping of the brain and captures either theoretical or actual positioning of the device (Fig 1E). In some cases, these images may also inform titration of acoustic energy to match dose across subjects. Among the various tissue types, skull bone is the primary source of acoustic wave distortion and absorption, and considerable variability exists in skull properties across individuals [47,48]. Thus, personalised skull imaging and acoustic simulations of losses are critical for optimal TUS interventions (Fig 1F) [44].

Although careful planning and simulation is common practice for scalable and reproducible TUS research, methods for validating targeting post-planning exist. MR-acoustic radiation force imaging (MR-ARFI) quantifies the small displacements of tissue generated at the ultrasound focus within the brain, and thereby provides direct confirmation of the mechanical impacts of TUS. Indeed, MR-ARFI uses the inherent property of TUS being propagated as a longitudinal wave. As the waves get closer to the focal zone, interference patterns lead to increased wave amplitude and resulting physical displacement. MR-ARFI uses motion encoding gradients that are applied simultaneously with the onset of the TUS pulse to image the peak displacement and, thus, the focus [49,50]. Although not yet commonly used in TUS research, MR-ARFI will be able to enhance the safety and efficacy of TUS interventions by enabling clinicians or researchers to monitor tissue interactions in real-time and adjust TUS parameters accordingly. Similarly, MR-thermometry can be used to measure focal temperature rise, a method currently employed in high-intensity focused ultrasound ablation. DBS electrodes can also be used to detect tissue displacement and may be leveraged to confirm target engagement [51,52].

## TUS: A search and rescue tool

**Fig 2:**
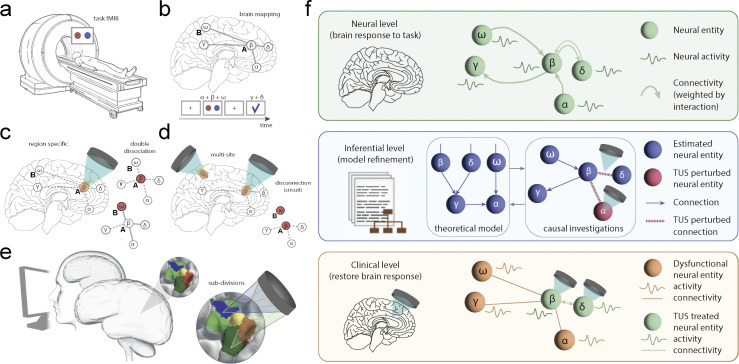
TUS in precision neuroscience: a search and rescue tool. (**a**) Participant in an MRI scanner performs a task. (**b**) Inferential level where brain mapping is made between cognitive processes and mechanistic features of brain regions function. (**c–e**) Causal manipulation can happen at the region level (c), allowing double dissociation intervention, (d) at the circuit level, allowing disconnection logic and the impact or circuit function or (e) at the subregion level, including subdivisions of anatomically defined brain nuclei. (**f**) Ontological levels relevant to TUS, defining the relationship between neural mechanisms (neural level) to inferences and modelling of brain target and circuits theoretical properties (inference level) to clinical interventional (clinical level). **Top panel:** At the neural level, a brain circuit mediates different cognitive processes (triggered exogenously or endogenously), denoted with Greek letters, within defined regions coupled with others. **Middle panel:** At the inferential level, theoretical models attempt to infer the relationship between behaviours and brain regions. This can be refined by causally manipulating brain regions with TUS to estimate the impact of brain perturbation on certain hypothesised cognitive processes. **Bottom panel:** Once an area is identified to relate to a specific cognitive process mapped onto psychiatric traits, this brain region can be perturbed with TUS to restore healthy functioning. MRI, magnetic resonance imaging; TUS, transcranial ultrasound stimulation.

Computational approaches of cognitive and underlying neural mechanisms have been a game changer for psychiatry [10]. In particular, task-performing computational models that explain how cognition arises from neurobiologically plausible dynamic components have been central in better understanding functions of the brain [11,53]. In a typical setting, participants engage in tasks designed to probe cognitive processes relevant for the disorder at play while undergoing neuroimaging, for example, fMRI (Fig 2A and 2F). The brain activity patterns observed are then associated with behaviour through computational modelling of cognitive processes usually timed to different onsets of the task (Fig 2B). The model features can then provide insights into which patterns of brain activity produce psychiatric traits. Despite progress, computational neuropsychiatry seems to have reached a plateau. Although data-driven studies allow clear visualisation of brain patterns, they lack interpretability and mechanistic insights. In contrast, theory-driven studies purport strong mechanistic foundations, but are difficult to test given the complexity of the human connectome in mental disorders [54]. In addition, most studies use neuroimaging techniques that remain largely correlational. As such, proof of true causality of computational parameters (e.g., features in decision-making or adaptability) in neuropsychiatric disorders is still lacking. Psychiatry urgently needs probing tools to offer mechanistic insights into mental health disorders while enabling direct causal claims and treatment strategies. TUS presents the potential for both, effectively serving as a “search and rescue” tool for the brain (Fig 2F).

Being able to make causal claims about neural computations with TUS has been achieved in nonhuman primates. For example, repetitive TUS has been found to disrupt activity in specific cortical and subcortical regions, perturbing their corresponding distributed networks. These perturbations directly influence behaviour, bringing insights into the causal role of several areas of the brain, including the function of the anterior cingulate cortex in translating cue information into decisions [42], the lateral orbitofrontal cortex in credit assignment [55], the basal forebrain in timing decisions [56,57], and the medial frontal cortex in estimating novel choice values [58]. In these studies, TUS applied specifically to spatially discrete brain regions allows examination of whether encoding of a specific behaviour is disturbed. If TUS perturbation leads to behaviour mirroring those observed within a distinct patient cohort, then causal claims may be suggested [59]. For example, increased prediction error and reward sensitivity, as seen in substance use disorder, can be achieved by perturbing the nucleus accumbens [60], a primary target for DBS in addiction [61,62]. This supports TUS as a method for identifying causal and therapeutically tractable brain targets [41,63].

Functional testing can be achieved at different levels of anatomical organisation. At the regional level, single or double dissociation logics have played a central role in testing the specificity of region–function relationships and are now possible in humans with TUS. A single dissociation logic can be used to test the causal relationship between a region A and a cognitive function (ω), whereas a crossover double dissociation can be used to make a stronger inference claim by demonstrating that region A is involved in a given function (ω) but not another (β), whereas region B is involved in β but not ω (Fig 2B and 2C). So far, this has been mainly limited to lesions studies, particularly involving animals, or limited to rare cases of circumscribed brain injuries in humans [64]. At the circuit level, testing the function of a particular neural pathway is possible by observing the consequences of disrupting communication between 2 or more brain regions from the broader neural network [65]. This approach allows for a more precise understanding of the neural circuitry underlying the function of interest, as it provides additional information on the role of information flow between regions (Fig 2D). Additionally, the possibility of multi-sites circuit sequence stimulation is especially important for biophysical modelling, which aims to understand both local and global circuit-level mechanisms in the brain (Fig 2D). Finally, as TUS technologies progress towards higher spatial resolution, TUS studies have started to look at subdivisions of nuclei interventions, for example, subdivisions of the striatum or other areas of the basal ganglia, in the context of psychiatric disorders (Fig 2E).

## Translating TUS to the clinic

Given the potential for TUS to identify causal regions in pathological behavioural outputs and its excellent safety reports, it is crucial to understand whether TUS can have a clinically meaningful behavioural impact. These impacts depend not only on the magnitude but also the duration of effects. Although the clinical translation of TUS research is still in its early stages, several promising studies in various conditions suggest that target-specific TUS can result in symptomatic relief.

Essential tremor, characterised by uncontrollable shaking of the hands, arms, and legs, is caused by abnormal activity in motor circuits of the brain including the thalamus, motor cortex, and cerebellum. Several studies aimed at alleviating these symptoms with TUS have been conducted, targeting the ventral intermediate nucleus (VIM) of the thalamus or the dentatorubrothalamic tract (DRT) that connect the cerebellum to the midbrain and thalamus. These have shown promise in decreasing tremor amplitude for at least 30 min post-TUS [40,66]. Intriguingly, in [40], the authors also identified a subgroup of non-responder participants, indicating the need for dynamic target exploration or deeper mechanistic phenotyping. Notably, one study [37] stimulated both the VIM and the DRT, suggesting that some combination or site selectivity may contribute to the strong effects observed. Thalamic TUS in patients in a minimally conscious state resulted in significant clinical improvement, as measured through observed responsiveness to external cues [67,68]. Meanwhile, a pilot study in epilepsy showed a reliable decrease in seizure frequency over the course of months following repeated TUS sessions [69,70]. In psychiatric disorders, precision targeting of the nucleus accumbens in individuals with substance use disorders resulted in substantial, long-term reductions in substance cravings lasting more than several months [41]. Preliminary evidence suggests that multi-site targeting across distributed circuits can also have clinical benefits. Therefore, an iterative search of putative therapeutic targets may be a viable strategy for personalised TUS treatment of depression [71]. Interestingly, TUS could also be applied to neuropathic disorders. Although the effects of TUS on clinically diagnosed pain disorders have yet to be demonstrated, healthy subjects have been reported to experience a decreased sensitivity to pain following stimulation of the dorsal anterior cingulate cortex [72,73].

Importantly, nearly all of the aforementioned studies lack a within- or across-subject control group and must be interpreted accordingly given auditory and somatosensory confounds of TUS [74,75]. Nevertheless, the diversity of clinical applications and brain areas targeted highlights the versatility of TUS. As research progresses, further studies with robust control groups and longer follow-up periods are necessary to fully understand and harness the potential of TUS in clinical settings. In the context of therapeutic approach to psychiatric disorders, it is important to highlight that TUS also offers a noninvasive, targeted method for modulating brain activity with fewer systemic side effects compared to pharmacological treatments, which often cause widespread effects and potential adverse reactions. It also allows for precise control over specific brain regions, facilitating tailored interventions that can be adjusted in real time.

## Challenges in TUS neuromodulation

TUS is rapidly gaining momentum in research and clinical use due to its portability and versatility, from treating to enhancing cognitive function. In addition, TUS equipment is relatively affordable, broadening its accessibility. However, challenges must be addressed for successful implementation.

Common to all neuromodulation technologies is the challenge of finding the most effective stimulation parameters (see Box 1). Similar complexities have been encountered in other neuromodulation techniques such as DBS or TMS [76,77] and have improved efficacy through the development of specialised pulsing protocols and accelerated delivery methods [78,79]. The multitude of parameters underlying TUS delivery, including pulse repetition frequency, intensity, duration, and spatial targeting, necessitates a systematic approach to protocol selection. To reduce the parameter space of TUS and enhance precision and safety, several strategies can be employed; these include implementing closed-loop control systems for adaptive tuning [80,81], directly measuring tissue temperature changes or mechanical changes with techniques like MR-thermometry or MR-ARFI, and developing patient-specific models for tailored stimulation. Such methods could minimise off-target effects and improve therapeutic efficacy in brain stimulation.

**Box 1 –Protocols and parameters for TUS**

TUS efficacy and safety are contingent upon the chosen stimulation parameters. Fundamental frequency, pulse duration (PD), pulse repetition frequency (PRF), duty cycle (DC), stimulus duration (SD), and intensity are among the important parameters that define a sonication protocol. The International Consortium for Transcranial Ultrasonic Stimulation Safety and Standards (ITRUSST) has provided comprehensive guidelines on stimulation parameters, best practices [44] and how to define TUS protocols [45], as well as recommendations for minimising both mechanical and thermal risks associated with TUS [46,82]. In short, TUS fields are generated by fundamental frequencies typically ranging from 250 to 1 MHz, dictating how quickly molecules oscillate. Related to the amplitude of the wave, the pressure determines the extent to which molecules deviate from their initial position, typically falling within the 0.3–1 MPa range inside the brain, while intensity is defined as power transferred per unit area with the average intensity of a pulse (spatial-peak pulse-average, ISPPA, described in Watt per cm2). TUS is commonly administered in a pulsed mode, with parameters such as PD and PRF within an SD spanning a few seconds to minutes.

In addition to finding the right dose, the field will need to determine the volume of tissue that needs to be sonicated for clinical effects, whether it be discrete areas of the brain, subregions within an area, or even a pathway between 2 regions or more complex circuits (Fig 2C–2E). This may depend on the amount of time it takes to observe TUS-induced readouts. Realistically, TUS may have delayed or cumulative effects which may make rapid spatial phenotyping either infeasible or unsafe. While sufficiently constrained fields may be searched in a reasonable timeframe, readout speed may be improved with molecular or biophysical enhancements to TUS. To improve the robustness of TUS, interdisciplinary collaborations and integration of advanced imaging techniques like MR-ARFI providing real-time feedback on tissue responses, or other forms of BMI closed loop paradigms, may also aid in the refinement of TUS protocols [81,83]. Additionally, large-scale research and clinical studies can help identify predictive biomarkers and optimise personalised treatment strategies. By leveraging these approaches, we can navigate the high dimensionality of TUS parameter space more effectively, leading to enhanced therapeutic outcomes and broader clinical adoption of TUS as a neuromodulation modality.

The challenge of clinical devices that do not rely on direct MR measurements (such as MR-ARFI), and potentially home devices in the context of advanced therapy, is verification of target engagement. Engagement has been a long-standing challenge in the TUS field, since pressure cannot easily be measured below the skull, and TUS-induced effects may have lagging or progressive onset. Nevertheless, inexpensive sensors can monitor features such as heart rate variability, electrical brain activity, galvanic response, pupil dilation, or motor coordination and balance. Using aggregate data may be used to grade target responses. As costs are reduced dramatically with emergent MRI technologies [84], pre-treatment imaging will become far more tractable for all TUS technologies and acoustic simulations will be guaranteed for each intervention.

Another challenge is the concept of state-intervention interaction, which highlights the dynamic relationship between the brain’s current state and the effectiveness of TUS [85]. It has been well established through noninvasive brain stimulation research that neural circuits may exhibit greater plasticity, or adaptability, under specific endogenously or exogenously triggered conditions [86,87]. For example, during certain cognitive tasks or heightened attentional states, neural circuits may be more receptive to modulation [88–90]. It has been shown that TUS exhibits greater efficacy and induces more significant circuit alterations and neurochemistry changes when TUS is targeted at active regions [43] and that TUS effects depend on the baseline regime of neurons [85]. This state-dependence could stem from various factors, including the release of neurochemical facilitators associated with the state or simply the physiological readiness of neurons to respond to stimulation. Irrespective of the specific mechanisms at play, this phenomenon highlights the necessity of considering the individual endogenous state or context when intervening with TUS.

## Towards the future of TUS

While early therapeutic TUS may be entirely relegated to clinical settings with highly regimented treatment strategies and technician operations, more complex use-cases will likely develop over time. However, the trajectory of these developments relies heavily on the incremental success of early approved indications. Essential tremor treatment has been a major target of DBS, the second largest cohort of patients after Parkinson’s disease, with over 160,000 surgeries performed to date [91]; its uptake is largely owed to easily quantified reductions in hand tremors and well-defined brain targets [92]. For the same reasons, disorders with similar features could also serve as landmark indications for ultrasound neuromodulation and are currently being investigated in registered clinical trials [93]. Once established, use of TUS may advance to severe cases of psychiatric issues for which DBS has shown some success [94–96]. Although these disorders pose greater challenges due to their multifaceted nature, the steerability of focused ultrasound holds potential for precise multi-site and tailored interventions. For example, stereotactic DBS site testing can identify the most efficacious target as performed with DBS [9]. Even in well-understood circuits, small differences in target positioning may produce markedly different effects [40]. Thus, the marriage between functional testing and therapeutic delivery may eventually serve as an integral component to therapeutic TUS translation. With early indications focusing on therapeutic interventions, it is plausible that TUS may extend beyond medical applications to enhance cognitive function in healthy individuals [97]. However, such endeavours necessitate careful consideration of ethical implications and rigorous evaluation of safety and efficacy [98].

**Fig 3:**
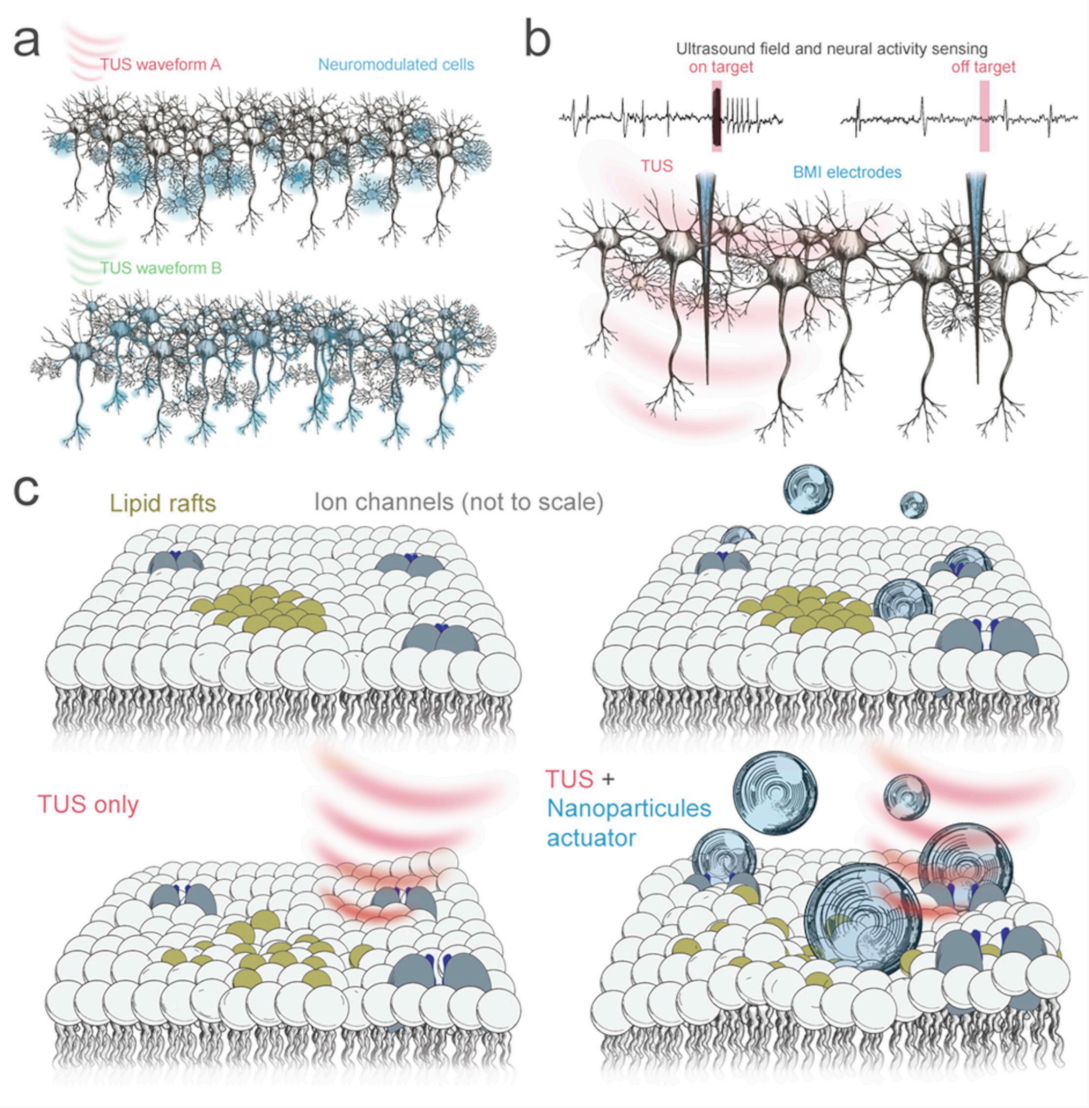
Conceptualizations of next generation TUS innovations. (**a**) Cell-type biased neuromodulation through application of variant TUS waveforms. (**b**) Illustration of BMI electrodes recording local field potentials during TUS exposure. Note the potential mechanical “artefact” recorded during the TUS delivery window which may be used to validate that the target is being exposed to ultrasound. (**c**) Illustration of lipid rafts within the neuronal membrane. Comparison of lipid raft and membrane disruption with TUS only (left), compared to TUS with microbubbles which expand during the low-pressure phase. Microbubbles may enhance mechanically derived neuromodulatory effects. BMI, brain–machine interface; TUS, transcranial ultrasound stimulation.

**Fig 4:**
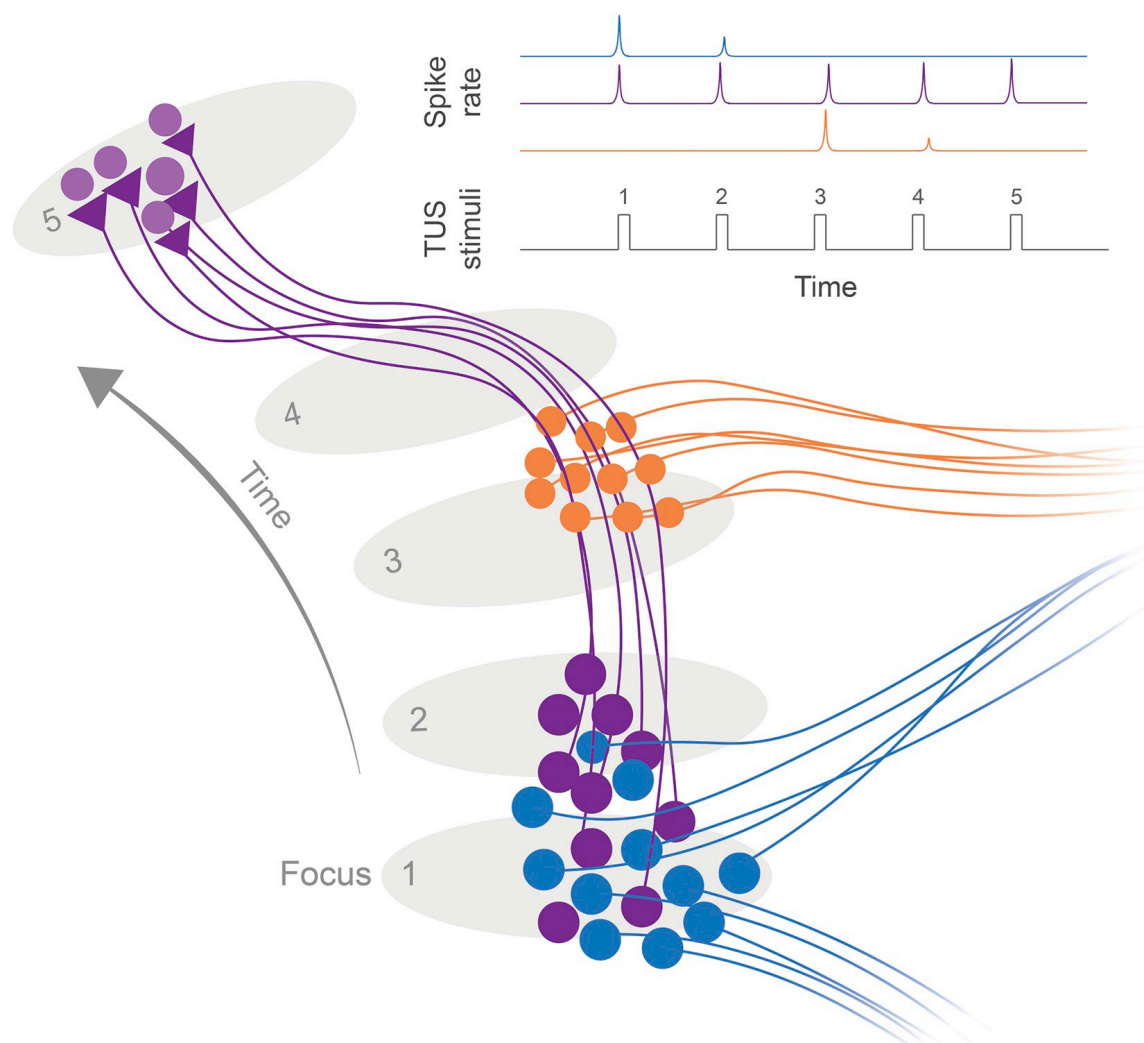
Circuit tracing as a method of cell type selective neuromodulation. A graphical illustration of 3 partially overlapping cell types shows how a focus can be moved along the tracts of a target neuronal circuit (purple). While cell bodies of off target populations may partially overlap at the targets cell bodies (blue) or axonal tracts (orange), exposure is limited to foci at a subset of raster positions. In this hypothetical scenario, only the purple cell type experiences repeated TUS induced spiking increases over the stimulation period. TUS, transcranial ultrasound stimulation.

With the constant improvement in our ability to monitor the activity of genetically defined cell types, future studies will likely continue to characterise cell-specific sensitivity to TUS. While the earliest evidence of cell type specificity come from peripheral nerve fibre type differences [99], recent works have highlighted differences in excitatory and inhibitory cell types across different brain regions, and their distinct sensitivity to variant waveforms (Fig 3A) [19,100]. These differences may be explained by intrinsic mechanosensory channel expression, membrane properties, and network interactions of various cell types [21,24]. Further refinement or discovery of efficacious waveform features that distinguish or elicit cell type biased responses will be critical to designing effective therapeutic strategies. Beyond sensitivity and pulsing to obtain cell type specificity, spatial anatomical characteristics of cell types may be exploited. For example, a select cell type may overlap with numerous others but may be spatiotemporally distinguished along its anatomy (dendrite, axon, and soma). By tracing the anatomy of the specific cell type, other overlapping cell types will having limited exposure (Fig 4).

In addition to neuronal sensitivity, several works have independently demonstrated that astrocytes may have heightened sensitivity to TUS [17,20,101]. Given that astrocytic activity plays a substantial role in modulating local neuronal activity, catering waveforms to astrocytes may be a viable strategy for lowering required pressures for treatment. From a future perspective, we can expect that high dimensional mapping of expression profiles across the brain may yield TUS sensitivity maps. More expanded brain interfaces mapping electrical activity to cell types may also expedite our understanding of TUS sensitivity.

Given the breadth of brain monitoring technologies currently integrated with TUS [102], it is worthwhile considering its combination with the rapidly expanding portfolio of brain–machine interfaces (BMIs) [103]. BMIs vary widely, from noninvasive systems like electroencephalograms, to invasive platforms such as multielectrode arrays (MEAs) and microwires. MEAs consist of rigid arrays of electrodes implanted directly into neural tissue, enabling precise recording and electrical field changes near or within neural tissue. Microwires, which are finer and more flexible, penetrate deeper brain regions with minimal disruption [104]. While these mechanical interfaces have not yet been combined with TUS, DBS leads offer a simplified model that could inform future integration with more complex BMI technologies. While human implanted multielectrode arrays or dense microwire platforms have not yet been combined with TUS, work with DBS leads might act as a simplified model of more complex BMIs. Classical DBS employs single or several electrical leads that can rest in cortical areas or extend into deeper brain structures. These leads are used to apply therapeutic levels of electrical current and are often capable of monitoring local field potentials [91]. While the use of TUS explicitly at the target may seem somewhat redundant or obsolete, voltage-gated and mechanosensitive channels may have only partial overlap in responsivity to either electrical or mechanical inputs [105,106]. Thus, it is plausible to expect that distinct and additive effects may be achieved by combining electrical and mechanical stimulation, albeit with careful optimization. In fact, recent work showed that ultrasound may enhance the signal strength of brain–computer interfaces (BCIs, which enable direct communication between the brain and external devices), and therefore decoding accuracy [107].

TUS may also be used towards brain areas not reached by existing BMIs. As is the case with several flagship devices, even thousands of electrical probes in the brain may still have access to only a small volume of cortical tissue [108]. Given the noninvasive nature of TUS, this therapeutic combination can be easily implemented with those who have already received implants. As lead count and spatial coverage continues to grow, these interventional strategies may be expanded to account for more complex network modulation. Beyond the combinatorial therapeutic strategies, BMIs may also serve to validate both TUS targeting and its effects. For example, TUS focused on the lead would certainly displace both the surrounding tissue and fluids, likely leading to heavy signal distortion (Fig 3B). This signal distortion strength may be related to focus-lead target distance and used to validate TUS brain targeting accuracy, since missing the target should result in substantially less distortion. Furthermore, changes in neural activity following TUS intervention may be observed as seen with mice [109]. Recent technologies combining functional ultrasound imaging (fUSI) with neuromodulation have been developed for animal models with thin skulls [110]. Although invasive, fUSI is being implemented over broad areas of the human brain by replacing skull fragments with imaging arrays which may also be used therapeutically [80]. Its resolution will vary depending on the transmit wavelength, which is not limited by the bone absorption observed with higher frequencies. More recent works are demonstrating large blood vessel Doppler imaging with relatively lower frequency. These methods offer “read write” functionality—referring to the ability to both monitor brain activity (“read”) and actively modulate it (“write”)—akin to traditional BCIs, although the dissociation of typical neurovascular coupling observed under some forms of ultrasound neuromodulation may complicate interpretation [100].

Although TUS has many benefits over traditional neuromodulatory approaches, its effects at shorter time scales are seemingly less potent than the use of directly applied electrical or electromagnetic fields [15,21]. To date, induction of simple motor events through cortical stimulation, common to TMS, have not yet been clearly demonstrated in humans, although multiple smaller animals studies have shown its efficacy in inducing lateralised and precise motor readouts [111]. While new evidence is emerging that VIM stimulation with TUS might have comparable effects to DBS, the effect onset appears to occur minutes after stimulation [40]. Although pure TUS neuromodulation may be relegated to use cases where slower cumulative effects are suitable, there is a growing interest in the enhancement of TUS neuromodulation through introduction of exogenous molecules in various forms. Although not explicitly tested in the context of TUS, the anaesthetic propofol is known to lace into lipid rafts, increasing their fluidity and propensity for disruption. This, in turn, increases downstream biochemical channel opening, suggesting that TUS effects may also be enhanced through propofol administration [112]. Gas-filled nanoparticles and microbubbles commonly used in ultrasound imaging have also been introduced to in vivo models during focused ultrasound administration [112,113]. Greater levels of neuromodulation were observed with bubbles compared to a vehicle control. It is hypothesised that these particles may act as force transducers, creating indentations and displacing the neuronal membrane as they oscillate in size [114]. This mechanical force should theoretically result in greater kinetic disruption of the lipid membrane and constituent rafts, in turn increasing ion channel opening (Fig 3C) [27].

In addition to transiently introduced molecules, genes that express mechanosensitive channels can be engineered into targeted cell types [115–117]. This approach can encode stable ultrasound sensitivity, allowing for persistent ultrasound neuromodulation at lower pressures than traditionally required for neuromodulation. Delivery of these genes with spatial precision may also leverage focused ultrasound to release transgenic plasmids through the blood brain barrier into local tissues [118], and cell type specificity may be achieved through engineering of specific promoters. However, transgenic engineering of the human brain must overcome vast safety and regulatory challenges over the next decade before therapeutic applications.

**Fig 5:**
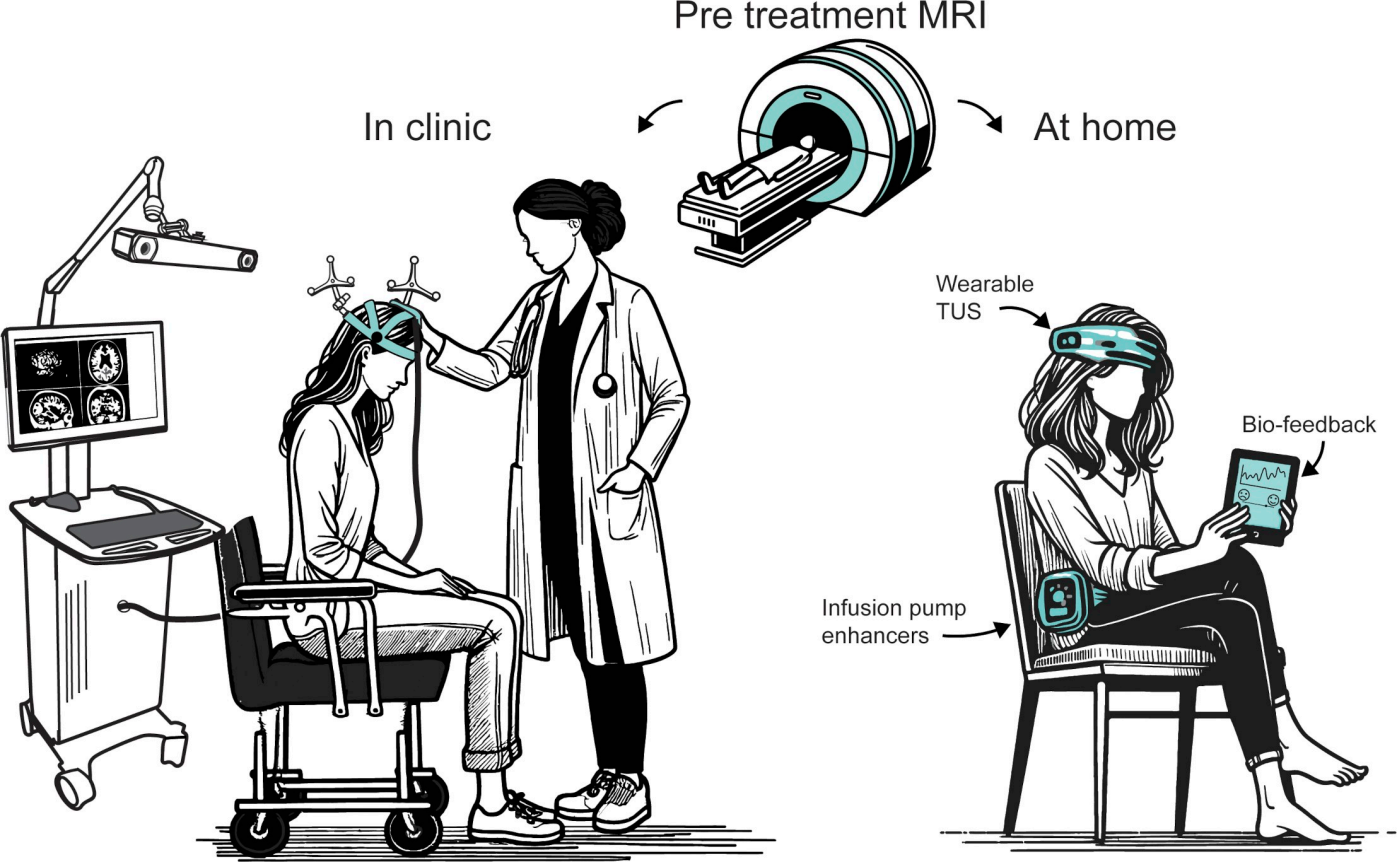
Conceptualisation of next generation TUS interventions. Clinical and at-home intervention are shown and involve meticulous verification of target engagement to ensure precision and safety. In-clinic use may include MRI to plan the intervention. Advances in simulation accuracy and quality control research will establish the reliability of TUS procedures. At-home TUS systems could be used for chronic symptom alleviation but require personalised MRI guidance and regulatory approval to ensure safety and efficacy. MRI, magnetic resonance imaging; TUS, transcranial ultrasound stimulation.

Aside from increased mechanotransduction, electrical currents may be induced through pressure-exposed piezoelectric materials, to mimic electrical lead DBS noninvasively. Boron nitride nanotubes exhibit piezoelectric properties and can convert acoustic pressure into small electrical charges [119]. Introduction of these molecules into neuronal cell culture caused significant increases in neurite outgrowth following ultrasound stimulus exposure, indicating underlying activity change. Similarly, piezoelectric tetragonal barium titanate nanoparticles introduced to neuronal cultures substantially improved calcium influx at low ultrasound pressures [120]. While much work is needed to identify the appropriate molecules and a means of repeated delivery, exogenous enhancers may reduce response latency and improve sensitivity, allowing for stimulus locked neuromodulation at lower pressures. For more severe psychiatric disorders, treatments supplemented with these particles through the use of a medical infusion device akin to an insulin pump could be envisaged (Fig 5). Although early use of these molecules will likely require highly controlled administration and monitoring in clinics, body worn pumps in conjunction with portable smart TUS systems may be possible given the vast success of body-worn insulin pumps and glucose monitoring.

Importantly, the first approved indication for TUS interventions will likely involve meticulous verification of target engagement. This will entail in-clinic assessments, such as MRI to plan the intervention and verify temperature changes or tissue displacement to ensure precision and safety. Advances in simulation accuracy and quality control research will be crucial in establishing the reliability of TUS procedures, paving the way for broader adoption and acceptance in clinical practice. Given the portability of TUS systems relative to larger NIBS equipment, it is also plausible that at-home systems may be used for chronic alleviation of symptoms (Fig 5). However, it is important to recognise the need for personalised MRI-guidance, or equivalently proven methodologies, to ensure safety and efficacy. Any at-home devices should be approved by the appropriate regulatory agencies as well as prescribed and carefully monitored by a physician for proper use. Unfortunately, the low cost and portability of TUS systems may lead to “do-it-yourself” efforts which pose safety risks and may be entirely inefficacious, an issue which has plagued the transcranial electrical stimulation field [121].

Acknowledging the state-dependence of TUS also suggests that some sort of facilitation could enhance the effectiveness of TUS interventions. By aligning stimulation protocols with specific behavioural states, such as engaging in cognitive tasks or maintaining a relaxed state, researchers may optimise the brain’s receptiveness to TUS-induced changes in neural activity. Moreover, understanding how different states influence neural plasticity could inform the development of personalised TUS protocols tailored to individuals’ unique cognitive and behavioural profiles. Exploring the integration of TUS with behavioural therapy presents a similar promising avenue. Upon identifying a network implicated in a particular disorder, activating a specific set of neurons within it through behavioural therapy enables the targeted modulation of this already therapeutically active neural system via neuromodulation. A proof-of-concept study for this type of functional targeting used TMS alongside concurrent working memory training to address working memory deficits resulting from sleep deprivation [122]. Ultimately, integrating knowledge of state-dependent plasticity into TUS interventions holds promise for enhancing their efficacy and paving the way for more targeted and efficient neuromodulation strategies.

## Conclusion

TUS offers a versatile approach for both exploring neural mechanisms underlying cognitive functions and providing potential therapeutic interventions for various neurological and psychiatric disorders. Despite challenges related mainly to parameter space and state dependency, the roadmap for TUS emphasises initial therapeutic applications in clinical settings, particularly targeting disorders with well-defined biological readouts with potential expansion to more complex psychiatric and neurological conditions. As the field progresses, considerations for cognitive enhancement in healthy individuals emerge, necessitating thorough evaluation of safety and efficacy alongside advancements in target engagement verification and procedural reliability. Integration with existing technologies, such as BMIs, and exploration of enhancer molecules, offer avenues for refining TUS interventions and enhancing its potential for clinical translation.

## Abbreviations

BCI: brain–computer interface
BMI: brain–machine interface
DBS: deep brain stimulation
DC: duty cycle
DRT: dentatorubrothalamic tract
fMRI: functional magnetic resonance imaging
fUSI: functional ultrasound imaging
MEA: multielectrode array
MRI: magnetic resonance imaging
MR-ARFI: MR-acoustic radiation force imaging
PD: pulse duration
PRF: pulse repetition frequency
SCC: subgenual cingulate cortex
SD: stimulus duration
tDCS: transcranial direct current stimulation
TMS: transcranial magnetic stimulation
TUS: transcranial ultrasound stimulation
VIM: ventral intermediate nucleus
